# 10Vida: A Mental and Physical Health Intervention for Chronically Ill Adolescents and Their Caregivers in the Hospital Setting: An Open Study

**DOI:** 10.3390/ijerph19063162

**Published:** 2022-03-08

**Authors:** Pilar Rodríguez-Rubio, Laura Lacomba-Trejo, Selene Valero-Moreno, Inmaculada Montoya-Castilla, Marián Pérez-Marín

**Affiliations:** 1 Department of Personality, Assessment and Psychological Treatments, Faculty of Psychology and Speech Therapy, Universitat de València, Av. Blasco Ibáñez, 21, 46010 Valencia, Spain; prr.psicologa@gmail.com (P.R.-R.); laura.lacomba@uv.es (L.L.-T.); inmaculada.montoya@uv.es (I.M.-C.); 2 Department of Developmental and Educational Psychology, Faculty of Psychology and Speech Therapy, Universitat de València, Av. Blasco Ibáñez, 21, 46010 Valencia, Spain; selene.valero@uv.es

**Keywords:** adolescents, chronic illness, family, psychological therapy, health

## Abstract

Suffering from a chronic disease (CD) in adolescence can significantly impact the emotional health of adolescents and their families. MHealth can be a useful tool for these groups. However, few intervention programmes include the family system. The aim is to design an intervention programme (10Vida) for a paediatric population with a CD, and their families, to improve their adaptation to the disease. The study is a quasi-experimental repeated measures design in a open study, where the patients themselves, and their families, are their own control group. Participants will receive an intervention of seven individual sessions: five sessions with each patient, and two sessions with their caregivers. In the case of the patients, the aim is to improve their emotional state, their self-esteem, and their emotional competencies, reducing their perceived threat of illness. Furthermore, in the case of the caregivers, the aim is to improve their emotional state and reduce their burden. Indirectly, working with caregivers and those being cared for will improve family ties. The pilot study will involve 25 to 30 chronically ill adolescents aged between 12 and 16 years and their primary caregivers. Following the results, the necessary modifications will be included, and the programme will be offered to adolescents and their families who are willing to participate.

## 1. Introduction

Adolescence is a period characterised by a multitude of changes at the biopsychosocial level, all of which also implies many challenges [1]. If the diagnosis or the presence of a chronic disease or condition (CD) is added, the adjustment of the adolescent to this period becomes even more difficult [2]. The World Health Organisation [3] defines chronic diseases (CDs) as “long-lasting and usually slow-progressing diseases’’. Among the main child juvenile CDs are the allergic, the endocrine (in particular, type 1 diabetes mellitus (T1DM) and short stature (SS)) and the respiratory, such as bronchial asthma (BA) [4,5]. A chronic disease is characterised by unforeseeable changes in the course of the disease, a reduction in physical capacity, changes in appearance, a prolonged dependence on medical specialists, continuous treatments, and the need for assistance [6,7,8].

The presence of a CD in adolescence is a possible risk factor for developing a psychological disorder and for decreasing physical and psychological well-being [9].The most common psychopathology in a childhood juvenile CD is emotional, particularly anxiety symptoms, followed by depression symptoms, possibly leading to the development of an anxiety disorder or a major depressive disorder, although behavioural problems are also common [10,11]. However, the adolescent’s perception of their illness may mediate this relationship. Thus, adolescents with a higher perceived threat of illness tend to show poorer physical and emotional health outcomes [12,13,14].

The disease affects not only adolescents, but also their entire family system. When someone is diagnosed with a CD, the family as a whole is affected by the stressors associated with the disease and the side effects of treatment. There is an inevitable alteration to the whole family system, especially in cases where the patient is an infant or adolescent [7,15]. It is necessary to underline that studies indicate that most of the care of adolescent patients generally is undertaken by one specific member of the family, usually called the main caregiver [16]. Therefore, continued exposure to medical care, accompanying adolescents during medical visits, missing days of work, or even changing roles within the family can lead to overburdening or emotional problems for the main caregiver, such as anxiety or depression [17,18,19].

The stress due to the care tasks has been associated with anxiety and depression symptoms, often causing emotional disturbances in the caregivers, associating the above with greater emotional symptomatology in adolescents and a worse control of their disease [6,20]. However, as indicated above, the family is the main source of support when dealing with the disease. Therefore, factors such as family dynamics or attachment may favour the psychological adjustment of adolescents and their families [7]. Thus, social and family variables are fundamental in explaining emotional adjustment in adolescence [21]. Similarly, the enhancement of psychological strengths and virtues can be helpful in buffering the effects of stress [22,23]. Therefore, personal strengths are traits that are shown in the person over time that can help us to achieve well-being, which can be trained [24]. In this sense, programs aimed at their potentiation have focused especially on increasing social skills, openness to experiences, creativity, kindness, love, self-regulation, gratitude, and humour [25]. These programmes may be useful in addressing physical and emotional health in adolescents with CDs.

According to the American Academy of Paediatrics, medical treatment is insufficient for chronic paediatric diseases. There is a need to improve the child’s well-being or health-related quality of life, empowerment, and transition to healthy and productive adulthood. Many interventions in chronic diseases have been implemented in the scientific literature and have proven beneficial [26,27,28,29,30,31]. Generally, interventions have been based solely on the patient or the caregiver, with few interventions working together with both parties, as in the present study.

Typically, the types of interventions in this population have been characterised by educational techniques focused mainly on explaining the disease (psychoeducation) [32,33,34,35,36] and improving adherence. Clinical guidelines suggest a cognitive–behavioural approach to the psychological management of chronic diseases. It is essential to work with patients on coping strategies to help them deal with their symptoms, as well as disease care to improve their adherence to medical treatment and a better adaptation to the disease [37,38,39]. However, recent studies have shown how other psychological interventions, such as mindfulness [40,41], can improve the quality of life of adolescents with a chronic disease. Both at the individual and group levels, such interventions help manage the stress that negatively affects self-care behaviours and disease processes.

On the other hand, according to Iglesias [42], positive psychology has useful tools for controlling the disease, focusing on the individual’s psychological work for the emotional and physical stability of the patient. The most effective way to support them is through a safe and secure environment in the face of the disease. Interventions for CDs should be aimed at increasing self-confidence and self-efficacy, improving an optimistic explanatory style, and developing active coping strategies [43,44]

Finally, health interventions are currently being implemented through digital and technological platforms in patients with chronic diseases. The emergence of new technologies has provided a variety of tools that can be used, especially in the current COVID-19 pandemic situation [45,46]. MHealth is defined as “the practice of medicine and public health supported by mobile devices such as telephones, patient monitoring devices, digital assistants and other wireless devices” [47]. MHealth is a particularly useful tool for medical and psychological interventions in adolescents with CDs [48,49,50,51,52], especially as many need to minimise their contacts with other people [53]. Therefore, mHealth can be an opportunity to improve social relationships in this group, as well as increasing their social support, while working on improving physical and emotional health [10,48,49,50,51,52].

There are many programmes on psychoeducation with aspects related to the understanding of the disease, the symptomatology, the treatment, and the consequences of non-adherence. However, specific programmes on the emotional aspects of the acceptance of the disease in everyday life, as well as learning coping strategies, are needed for patients as a learning opportunity for the improvement of psychological well-being [26,27]. Psychological interventions in this area have primarily focused on the following components: disease beliefs, self-image and self-esteem, coping strategies and emotional competencies, and social support [37,44,54,55,56]. Of all the variables that have been focused on the different programmes, among the most important is the subjective perceptions or beliefs of the patient and their family regarding their illness [37,54,55], which can be an obstacle to treatment adherence. Another variable that has been of primary focus is self-esteem, which has been observed to be lower in adolescents with CD [44,56]. Considering the protective factors that a positive self-concept has on adjustment to stressful situations, different studies address the need to incorporate this variable as an outcome measure in intervention programmes [57]. In addition, as mentioned above, chronic illness at this stage can have a negative impact on the emotional well-being of adolescents. Therefore, working on emotional competencies, as previous studies indicate [26], is beneficial in reducing the emotional impact of the illness and fostering motivation to participate in self-care tasks. Finally, social relationships can be a protective factor, facilitating the adjustment to the disease [56]. It is necessary to assess their psychological adjustment and provide them with tools and support to participate in social activities without being prejudiced by their disease.

For interventions with families, the main focus has been to reduce the caregiving overburden [18,20,58] and allow for emotional ventilation, which contributes positively to a better adaptation to the disease by the patient [7,19].

To date, there are few intervention programs focused on adolescents with a CD and their families, and even fewer if they are approached through mHealth, an essential tool, given the global situation, that is on an individual basis to meet individual needs. That is why this research aims to (1) design an intervention program aimed at enhancing the strengths of adolescents with a CD and their families; and (2) analyse the effectiveness of this program on the indicators of the medical control of the different CDs (glycemic control, densitometry, analytical controls, spirometry, plethysmography, and the prick test) and well-being immediately after completing the training. The hypothesiss of the study is that adolescents will show a maintenance or improvement in physical and emotional health indicators after the implementation of the program.

## 2. Materials and Methods

Following the standard protocol items: recommendations for interventional trials statement, the current study protocol describes the details of the study rationale, proposed methods, organisation, and ethical considerations.

### 2.1. Study Setting

This study will be conducted in the different leading hospitals of the Valencian Community, in the Pneumology, Endocrinology, and Allergology units. This study is a quasi-experimental design with repeated measures. Each participant receiving the intervention (experimental condition)will also act as their control grorup. Therefore, the physical and psychological diagnostic measures will be collected from all of participants in an initial assessment (T1) and, secondly, six months after the initial assessment and right before starting the treatment programme (T2). At these two assessment points (T1 and T2), all participants (patients and family members) will be assessed (T2). The treatment lasted for five sessions for adolescents, with one individual session per month and two sessions for family caregivers (month 4 and month 5). The treatment programme will begin in the maximum expected time of 15 days from the second pre-treatment assessment (T2), with an estimated duration of 5 months. After the end of the treatment sessions for the patient and their family members, a new diagnostic test (T3) will be conducted (across all variables), within an expected maximum time of 15 days until the end of the treatment, to assess the post-treatment change. Thus, the estimated time between T1 and T2 is the same as that between T2 and T3, which is six months. The programme of registration, interventions, and evaluations (SPIRIT Programme) is shown in Table 1 and Table S1. 

### 2.2. Patient and Public Involvement

Participants will be recruited when receiving hospital care. Inclusion criteria will be (1) participants must be adolescents from 12 to 16 years of age, with (2) a diagnosis of CD (endocrinologic, such as DM1, or pneumo allergic diseases) within the last 6 months. Exclusion criteria will be (1) the presentation of a psychiatric disorder prior to the disease; (2) epilepsy, infantile cerebral palsy, or brain tumours; and (3) not understanding the Spanish language. All the parents of recruited adolescents will be offered the opportunity to participate in shorter sessions to promote self-care and family bonding. The parent training will be conducted in two 2-h sessions. The trainers will be psychologists specialised in health psychology who, prior to the start of the program, will receive hours of training in the 10Vida program.

### 2.3. Interventions

10Vida is a program that consists of different psychological objectives to be applied to adolescents between 12 and 16 years of age who suffer from a chronic disease thatincludes the pneumo allergic or endocrinological specialties. It is a 7-session intervention programme (five individual sessions with adolescents, and two sessions with family caregivers) consisting of one session per month with patients, and two sessions at the end of the treatment with family caregivers (month 4 and month 5).

**Figure 1:**
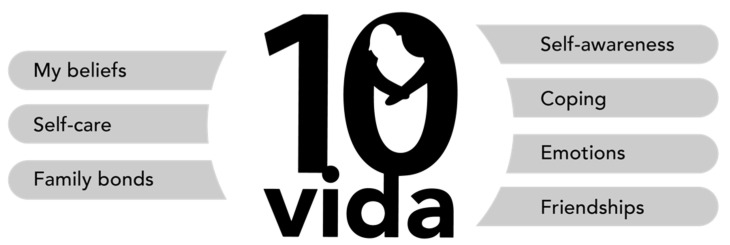
Presentation of the 10Vida programme and its areas.

It is a program that provides strategies and tools, trying to provide answers to the needs of the adolescent and caregivers, which can facilitate adherence to treatment and, therefore, improve physical and psychological health. It has been shown that a psychological intervention is necessary in addition to the important medical treatment, as it improves the emotional well-being of the adolescent and their families. 10Vida consists of individual sessions for both adolescents and their families, such as parents as caregivers who may be overloaded with tasks, worries, and the need to help their child to be happy. The specific objectives of each of the adolescent and caregiver sessions, as well as the areas to be worked on, are shown in Table 2. In addition, a summary of the strengths promoted by the programme are shown in Figure 1. To work on the different session objectives, various activities or tasks have been developed with different objectives to improve each of the key areas of the sessions. All activities can be found in Table 3. Participants who do not complete 80% of the sessions will be excluded from the study.

### 2.4. Outcomes

All instruments will be completed by the participants during baseline (T0, control), pre-intervention (T1), and post-intervention (T2).

Primary patient outcomes

- *Emotional adjustment:* Hospital Anxiety and Depression Scale (HADS): For the study of anxiety, depression, and global emotional distress [59] (scale validated for the Spanish adolescent population) [60]. In terms of psychometric properties, the reliability scores in the study with adolescents were 0.80;
- *Emotional competence:* Emotional Skills and Competence Questionnaire (ESCQ-21): This is a self-report measure developed by Takšić [61] to assess emotional competence. In the present study, the reduced version (ESCQ-21) was used, which was adapted and validated to a Spanish sample by Schoeps et al. [62]. Psychometric properties are adequate (perception and comprehension, α = 0.82; expressing and labelling, α =0.90; and management and regulation, α = 0.77);
- *Perceived illness threat* (BIP-Q) [63,64]: Assesses the patient’s perceived level of disease threat with their symptom control, associated emotions, the duration of the disease, and the causes or factors of the disease. Psychometric properties are adequate (α = 0.76);
- *Self-esteem:* Rosenberg Self-Esteem Questionnaire (RSE) [65]: Self-esteem was assessed using the Spanish version of this scale [66]. In terms of psychometric properties, the reliability scores in the original study were 0.92;

Primary caregiver outcomes

- *Emotional adjustment*: Hospital Anxiety and Depression Scale (HADS): for the study of anxiety, depression, and global emotional distress [59]. Regarding the scale’s internal consistency, a validation study in a Spanish adult population found that values for the anxiety scale ranged between 0.68 and 0.93, and for the depression scale, between 0.67 and 0.90;
- *Burden or stress*: The Paediatric Inventory for Parents (PIP) [6] to assess levels of caregiving-related stress [6,67]. It aims to assess parents’ stress with children requiring regular medical care. It consists of 12 situations related to the hospital environment that are considered potentially stressful for parents with ill children. Optimal reliability coefficients were obtained, with 0.78 for the total frequency scale and 0.81 for the total stress scale [6].

Secondary patient outcomes

- *Adaptation to chronic disease:* The Chronic Respiratory Disease Questionnaire (CRQ-SAS) [68] assesses aspects related to the adaptation to the disease (scale validated for the Spanish adolescent population by Valero et al. [69]). The psychometric properties are adequate (α = 0.85). The questionnaire on the adaptation to type 1 diabetes (RAE) [70] assesses aspects related to the adaptation to the disease, such as severity, adherence, psychological impact, discomfort, and health behaviours. Psychometric properties are adequate (α = 0.77). Finally, the Adolescent Rhinoconjunctivitis Quality of Life Questionnaire (AdolRQLQ) [71] is a quality of life assessment instrument specifically designed for the paediatric population with rhinoconjunctivitis. This questionnaire shows adequate psychometric properties (α = 0.77);
- *Parenting style of parents*: The Scale for the Evaluation of the Educational Style of Parents of Adolescents (EP) [72] allows for the assessment of the perception that adolescents have of their parents’ educational style;
- *Psychological well-being*: The Psychological Well-Being Scale for Adolescents (BIEPS-J) is a self-report measure developed [73] to determine the level of psychological well-being in adolescents (scale validated for the Spanish population [74]).

In addition to the psychosocial variables, objective measures, such as those obtained through medical indicators, were considered as secondary variables:

- *Glycaemic control:* Glycosylated haemoglobin (HbA^1^c) will be used to assess the glycemic control of the diabetic patient;
- *Somatometry to assess nutritional status:* Weight, height, body mass index (BMI), the brachial perimeter, and the tricipital fold will be used, with the calculation of percentiles and the z-score. To assess nutritional status, weight-for-height percentiles, BMI, or z-scores will be used. The aim is to maintain weight-for-height or BMI at the 50th percentile, as well as height/age (which is the best sign for disease control);
- *Spirometry values:* This will be used to determine pulmonary function. Spirometry is a test that studies pulmonary functions under controlled circumstances, as well as the absolute magnitude of lung volumes, and the speed with which the patient can mobilise them. The main spirometric parameters recorded are forced vital capacity (FVC), vital capacity (VC), peak expired air volume in the first second (FEV1), and peak expiratory flow (PEF);
- *Analytical controls:* This will be used (a) for monitoring nutritional status (protein, lipids, fat-soluble vitamins, iron and calcium-phosphorus metabolism) and Quick’s index; (b) for monitoring exocrine pancreatic function (faecal elastase and steatorrhoea); (c) for monitoring glucose metabolism; (d) for monitoring acute phase reactants and the immune status (immunoglobulins and alpha1 antitrypsin; and (e) for monitoring hepatic and renal functions;
- *Control images:* Using computerised axial tomography (CAT) or X-rays, the aim was to control the evolution of the disease through the appearance of bronchiectasis (small scars in the lung) or other types of pulmonary affections.
- *Others* (according to age): a) Plestimography values, whichdetect static lung volume, and b) bone densitometry, when there are risk factors for skeletal demineralisation.

Secondary caregiver outcomes

- *Resilience:* Connor–Davidson Resilience Scale (CD-RISC-10) [75] to assess the ability to cope with stress and adversity. The CD-RISC-10 is a shortened version of the CD-RISC [76];
- *Family functioning:* The Family Cohesion and Adaptation Scale (CAF-R) (FACES) [77]) will be used. It was developed to construct a measuring instrument to evaluate the variables that make up the dynamics of a family, based on Olson’s Circumplex Model. The scale consists of two dimensions: cohesion, understood as “the emotional bond that exists between family members” and family adaptability, that is, “the family’s capacity to change and adapt to change”.

All these variables would be assessed if sufficient participants were obtained. If not, the researchers would only use the primary variables for statistical analyses.

### 2.5. Participants’ Timeline

The first pilot study started in February 2019, following the proposed structure with a small sample of six participants (adolescents and their families), and it was concluded in January 2020. Due to the pandemic, this protocol had to be adapted to a fully online format while maintaining the activities and variables studied. Currently, the February 2022 participants are being recruited for a second pilot study to test the intervention and improve the programme. The main study is scheduled to run from September 2022 to September 2024. Table 1 shows the timeline for the enrolment, interventions, and evaluations. The main objectives of phase I are the selection of the hospitals, the request for informed consent, and the baseline assessment. Firstly, the services of the hospitals that the research team has convened will be contacted. After meetings with the directors, informed consent will be requested from the families and adolescents participating in the programme. Before implementation, patients will be asked to complete a set of questionnaires. Phase II’s main objective is to evaluate the participating patients and family members, as they will constitute the control group. After six months, in phase III, the main objective will be implementing the intervention programme in the same sample as in phase II. After completing the intervention, in phase IV, participants will be re-evaluated. In the last phase, the effectiveness of the programme will be analysed.

### 2.6. Sample Size

The initial idea is to perform the first test on a pilot sample of 25–30 patients from each unit, with which the investigators have collaboration agreements. This pilot study is intended to evaluate the quantitative data to assess whether or not there is an improvement. At the end of the intervention, a questionnaire will be conducted through LimeSurvey (an anonymous survey platform) with open-ended questions, such as: ‘What did you like most? How do you feel after the intervention? What improvements would you suggest? What would you like to change?’ The aim is to find out patient satisfaction and to suggest improvements for the final programme. Several members of the research team will carry out the analysis of the systematic qualitative data in order to achieve a consensus.

Subsequently, modifications will be made to the programme based on the results obtained, and the programme will be applied to those adolescents and their families who regularly attend hospital outpatient clinics and meet the inclusion criteria. Once implemented in a pilot sample, this protocol will be extended to a larger sample, with a sample size of approximately 200 chronically ill adolescents and their families.

### 2.7. Recruitment

Endocrinologists, pulmonologists, and allergists will be responsible for recruitment, which will take place in the hospital during routine patient visits. Participants will be asked to complete a battery of questionnaires on their psychological and physical health statuses in a single session. The design of this intervention consists of patients and caregivers who receive the treatment program, who will act as their own control group; then, after six months, the 10Vida intervention will be initiated. The outcome measures will be collected by the medical and psychological specialists.

### 2.8. Methods: Data Collection, Management, and Analysis

Assessments at baseline, pre-intervention, and post-intervention will be performed by the medical specialists and trained psychologists working together. The following sociodemographic and clinical variables of the children and adolescents will be collected by means of an ad-hoc registry: their date of birth, age, sex, and school year. Moreover, for the main family caregivers, the following variables will be collected by an ad-hoc registry: relationship to the patient, age, and presence of chronic disease in the caregiver.

Clinical variables will also be recorded (among them, and when applicable): the main diagnosis, secondary diagnosis, time of diagnosis, time of treatment, number of hospital and/or emergency room admissions, and the total number of these admissions that are directly related to the chronic disease, as well as the type and amount of medication taken, and frequency of regular medical control visits.

#### Statistical Methods

Independent-samples *t*-tests and chi-square tests were performed to determine pre-intervention differences between participants with and without missing values in the variables studied. In addition, chi-square tests were undertaken to compare the number of people who dropped out of the research in the experimental group with those who dropped out in the control group, and it was found that they did not differ significantly.

Before testing the change models, descriptive analyses, Pearson correlations, and multivariate and univariate variance analyses (MANOVA and ANOVA) were performed with the pre-intervention scores of the participants in the experimental and control groups to identify possible differences in the baseline. Likewise, multivariate and univariate covariance analyses (MANCOVA and ANCOVA) were performed to identify changes in the post-intervention scores (short-term effect), while controlling for the pre-intervention scores (covariate). In addition, the effect size (Cohen’s *d*) of each variable was calculated to estimate the magnitude of the differences between the experimental and control groups. These analyses were performed with the statistical package SPSS V.26.

Multiple hierarchical regression analyses were conducted to examine the added predictive value of the experimental condition to explain the effectiveness of the intervention programme [46]. Firstly, the pre-intervention assessment score (control variable) was introduced, and then the experimental condition (1 = experimental intervention group, 0 = control group) was introduced as an independent variable. The dependent variable was the change variable between pre- and post-interventions, as well as between pre-intervention and the follow-up of the emotional and well-being variables. In this sense, the statistically significant change of the determination coefficient in model 2 (predictor: experimental condition, controlling for the pre-intervention score) is interpreted as an added predictive value. The significant prediction of the experimental condition, controlling for the pre-intervention score, leads us to attribute the significant change to the intervention. All data shall be entered electronically. Participant files will be recorded securely and will be stored for a period of 10 years after the end of the study.

### 2.9. Ethics and Dissemination

Each participant will receive information about the aim and procedures of the study and will provide written consent for participation. Consent and assent will be obtained by the medical specialist. The data will be confidential and anonymised, and will be used solely for the objectives of the study (27 April 2016 Data Protection Act (GDPR)). Numerical codes will link each participant’s identification information. The data collected will be stored in a locker in the place of work of the principal investigator, and electronic data will be protected by password on the university network computer. Any protocol amendments will be registered at ClinicalTrials.gov (NCT04476433).

## 3. Discussion

The present study aims to design a strengths-based intervention programme for adolescents with a CD and their families. The expected results of this study could incorporate improvements in routine clinical practice. This programme will provide an effective tool to improve physical and emotional health. The design of this intervention programme has been based on previous psychological interventions [26,27,30,31], encompassing different components explored in the different research studies. However, one advantage is that it incorporates the caregiver in the programme, and it is carried out on an individual basis.

The second objective of the study is to assess the efficacy of the programme on indicators of physical and emotional adjustment immediately after the programme. We expect that adolescents will improve their personal strengths and, therefore, there will be an increase in their emotional skills, and their well-being, as well as an improvement in their social and family relationships and their quality of life, and a decrease in psychopathology. Similarly, we expect some medical indicators (glycaemic control, densiometry, analytical controls, spirometry, plethysmography, or the prick test) to improve or remain stable, as other programmes targeting adolescents with CDs have shown [30,31].

At the same time, including family caregivers in the intervention can provide us with an improvement in emotional health indicators and their personal protective factors, which can directly influence the physical and emotional well-being of their children [26].

The results after the implementation are expected to show the success of personal strengths enhancement, underlining the importance of family system-based interventions to enhance personal skills and treatment adherence in adolescents with CD [26,46]. Therefore, our intervention programme will consider primary caregivers in its sessions, dedicating two of the seven sessions to the management of the CD in the family setting. We believe that this intervention may be more effective than interventions specifically targeting adolescents, without taking their families into account [26].

The preliminary findings obtained from the current pilot study on adolescents with type 1 diabetes mellitus and their caregivers suggest that the 10Vida program provides an opportunity to enhance the physical and psychological adjustment of adolescents with a chronic disease, as well as their families.

Despite the strengths and potentials of the study, especially its multicentre scope and its ability to adapt to different pathologies suffered by adolescents, which will allow for the comparison of results between groups, this study has certain limitations. The fact that the control group is a waiting list control group may mean that the results are not directly attributable to the intervention programme. In addition, the samples we intend to access are difficult to access, and the study will not compensate participants financially, so the number of participants may be small. Nevertheless, the team will try to encourage the motivation of adolescents and their families to participate, and will widely disseminate the programme to try to reach as many participants as possible.

## 4. Conclusions

To our knowledge, this is the first programme specifically aimed at increasing the personal strengths of adolescents with endocrine, respiratory, or allergic CD, which also considers their families. Although an intervention protocol addressing emotional skills in adolescents with type 1 diabetes mellitus exists [49], no results are known to date. In addition, most intervention programmes are aimed at reducing psychopathology, but do not assess the enhancement of the protective factors in the family. Enhancing personal strengths in the families of adolescents with CDs may help them to better adapt to the disease and, therefore, manage it better. This may improve the course and development of CDs and prevent future medical complications.

## 5. Patents

This intervention protocol is registered as intellectual property at the Universitat de València, under the registration number of UV-MET-202131R (Registered on 24 May 2021)

## Figures and Tables

**Table 1:** Time schedule for study period with phases and assessments.

	Enrolment	Allocation			
TIMEPOINT **	*T1 (Own control group)*	0	*Pre test (t_2_)*	*Intervention*	*Post test (t_3_)*
**ENROLMENT**					
Eligibility screen	X				
Informed consent	X				
Allocation		Not applicable			
**INTERVENTIONS**					
*“10Vida”* intervention (experimental group)			X	X	X
**ASSESSMENTS**					
**Primary outcomes of patients**					
Sociodemographic data	X				
Emotional adjustment	X		X		X
Emotional competencies	X		X		X
Perceived illness threat	X		X		X
Self-esteem	X		X		X
**Secondary variables of patients**					
Adaptation to chronic disease	X		X		X
Parental parenting style	X		X		X
Psychological well-being	X		X		X
Physical or clinical measure (i.e., Hba1c)	X		X		X
Satisfaction with programme					X
**ASSESSMENTS**					
**Primary outcomes of caregivers**					
Sociodemographic data	X		X		X
Emotional adjustment	X		X		X
Burden or stress	X		X		X
**Secondary outcomes of caregivers**					
Resilience	X		X		X
Family functioning	X		X		X
Satisfaction with programme	X		X		X

**Table 2:** 10Vida program summary. Sessions and objectives.

10VIDA PROGRAM		
Sessions with Patients (5)		
Name of Session	Theme	Aims
S1. Mis creencias	Adjustment to illness	Assess, recognise, and value beliefs, concerns, or fears related to the disease
S2. Una mirada a mi interior	Self-esteem/self-concept	To develop behavioural patterns that facilitate an adequate self-image and identity, without the stigmas of illness
S3. Desde la serenidad	Coping with fear	To learn to identify, attend to, and manage the anxious symptomatology associated with the life situations that a chronic disease in adolescence may entail. To favour a serene and positive attitude, knowing their own fears
S4. Las emociones: Mis amigas	Emotional self-regulation	Encourage a coping and resilient attitude to facilitate the acquisition of appropriate habits and behaviours. To promote positive emotions that can cushion the daily situations with the disease
S5. Una mirada al exterior	Social area	To reflect on the importance of friendships at this age, and that they are sources of support in the face of illness and treatment
S6. Me cuidas, te cuidas	Caregiver needs	Know and address the psychological and emotional needs of primary caregivers by providing them with strategies. Intervene in the beliefs and concerns regarding the disease, their child, themselves, or the family
S7. Unidos, sumamos	The family system	Emphasise the role of parents in coping with their child’s illness, reducing stress, and encouraging acceptance. Encourage a democratic parenting style

**Table 3:** Content of 10Vida activities.

Session	Theme/Variable	Activity	Activity Objectives
S1. Mis creencias	Adjustment to illness	A1.1. Well_lived chronic disease	Provide the adolescent a leading role and create a safe space for them to express themselves and grow emotionally Recognise the main concerns that generate fear and the place of their illness within them Express and recognise feelings, beliefs, and thoughts about their illness
		A1.2. Deal	To provide tools for decision making in their life beyond the disease Respond to their emotions and thoughts to guide them towards achieving their goals and coping with their fears
S2. Una mirada a mi interior	Self-esteem/self-concept	A2.1. I am and not my disease	Work on the importance of self-reflection Encourage learning to look at and discover oneself with serenity and affection Define self-esteem and self-concept Reflect on the role of the disease in one’s self-image
		A.2.2. Because I’m worth it	Self-awareness and self-discovery: strengths, weaknesses, and needs
		A2.3. I spoke nicely	Reflect on the importance of words and their effect on thoughts and action Work on positive language and the effect of negative thoughts
S3. Desde la serenidad	Coping with fear	A.3.1. I listen to my signs	Through an explanation supported by drawings, learn the difference between fear and anxiety Understand how fear/anxiety works Learn how to detect and identify signs of fear/anxiety in your body
		A.3.2. My monster and I	Give name, form, “life” to the “monster” to have more control and stop fearing it Training of self-regulation resources
		A.3.3. The eye outwards	Explain the mindfulness-based technique: mindfulness in everyday life Learn to discover and be amazed by their surroundings and what they may not have noticed
S4. Las emociones: Mis amigas	Emotional self-regulation	A.4.1. Emotions, my Friends	Define the concept of emotions, their functions, and their meanings To facilitate personal emotional identification and expression To train in emotional skills
		A.4.2. Emociometer	Continue working with emotions and their functions Increase emotional vocabulary Training in emotional skills
		A.4.3 D&D	Discover effective strategies for expressing emotions
S5. Una mirada al exterior	Social area	A.5.1. I’m not alone.	Understanding the influence of others in our lives To observe how the disease has affected relationships and social situations
		A.5.2. Social superhero	Demonstrate the main social skills for their stage of development and describe the skills with them Social skills training
		A.5.3. Colour glasses	Learning to perceive the world through a more positive language
S6. Me cuidas, te cuidas	Caregiver needs	A.6.1. It is in my	Ask parents about the influence of their children’s disease on their own lives Provide a space and a place for them to express their fears, worries, frustrations, etc.
		A.6.2. Welcome adolescence	Provide a perspective of adolescence as both a challenge and an opportunity rather than a risk Explore the changes in the adolescent developmental process
		A.6.3. Because I also exist	Working on the importance of self-care
S7. Unidos, sumamos	The family system	A.7.1. Yes to love	Reminding parents of the importance of the bond of attachment with their children To give specific hints on how to show affection at this age and according to their individual needs
		A.7.2. I control	Explain to parents the need to control their children at the adolescent stage Working on learning styles
		A.7.3. Living without whatsapp	Provide information on the quality and quantity of communication with an adolescent Provide keys to more positive communication, favouring warmth and minimising conflicts

## Data Availability

Preliminary data from this study are available on request.

## Supplementary Materials

The following are available online at https://www.mdpi.com/article/10.3390/ijerph19063162/s1, Table S1. SPIRIT 2013 Checklist: Recommended items to address in a clinical trial protocol and related documents *.
